# Inhibitory Effects and Mode of Action of Pure Eugenol Versus Clove Essential Oil on Key Phytopathogenic Fungi

**DOI:** 10.3390/ijms27115083

**Published:** 2026-06-04

**Authors:** Francisca Sempere-Ferre, Josefa Roselló, María Pilar Santamarina

**Affiliations:** 1 Departamento de Estadística e Investigación Operativa Aplicadas y Calidad, Universitat Politècnica de València, Camino de Vera s/n, 46022 Valencia, Spain; 2 Departamento de Ecosistemas Agroforestales, Universitat Politècnica de València, Camino de Vera s/n, 46022 Valencia, Spainmpsantam@eaf.upv.es (M.P.S.)

**Keywords:** antifungal activity, *Syzygium aromaticum*, eugenol, statistics

## Abstract

The use of natural products as alternatives to synthetic fungicides has gained increasing importance in crop protection. Among these, clove (*Syzygium aromaticum*) and its active compound, eugenol, are well known for their antifungal properties. However, it remains unclear whether the antifungal activity of clove is primarily driven by its major constituent, eugenol, or whether the whole essential oil exhibits greater or synergistic efficacy. Addressing this question is crucial for optimizing their application as biofungicidal agents; The chemical composition of clove essential oil was characterized using gas chromatography–flame ionization detection (GC-FID) and gas chromatography–mass spectrometry(GC-MS). The antifungal activity of the essential oil and pure eugenol (300 µg/mL) was evaluated in vitro against *Botryotinia fuckeliana*, *Rhizoctonia solani*, and *Verticillium dahliae* on potato dextrose agar (PDA). Mycelial growth inhibition was quantified, and data were analyzed using two-way analysis of variance (ANOVA) followed by Tukey’s honestly significant difference (HSD) test (α = 0.05); Eugenol exhibited higher antifungal activity than the essential oil across all tested species. *V. dahliae* was completely inhibited (100%) by eugenol, while the essential oil showed lower efficacy. Despite the high eugenol content (87.3%) in the oil, its reduced activity suggests that minor constituents may modulate overall antifungal performance. These findings demonstrate that eugenol is more effective than clove essential oil as an antifungal agent. This highlights that the biological activity of clove is largely driven by its major active component, providing key insights for the development of more efficient biofungicidal strategies.

## 1. Introduction

Phytopathogens fungi represent one of the major threats to global agricultural production, causing significant losses in economically important crops and compromising food security [1,2]. Traditionally, the control of these organisms has relied on the use of chemical pesticides, the prolonged application of which has led to resistance issues, negative environmental impacts, and residues in agricultural products [3].

Species such as *Botryotinia fuckeliana*, *Verticillium dahliae*, and *Rhizoctonia solani* exemplify the diversity and impact of phytopathogenic fungi on agricultural systems. *Botryotinia fuckeliana* (anamorph *Botrytis cinerea*) is a necrotrophic pathogen responsible for grey mold, a disease that affects over 586 genera of vascular plants, representing over 1400 ornamental and agriculturally important plant species causing significant pre- and post-harvest decay in fruits, vegetables, and ornamentals worldwide. Its broad host range and ability to survive on senescent or dead plant material contribute to persistent disease pressure and economic losses across multiple cropping systems [4,5].

*Verticillium dahliae* is a soil-borne pathogenic fungus that induces vascular discoloration and wilt symptoms across a broad range of host plants, affecting approximately 400 species, including cotton, potato, watermelon, cucumber, and spinach. In 2021, it was estimated that *V. dahliae* reduced cotton yields in China by 15–20%, highlighting its significant impact on crop productivity and the challenges it poses for sustainable agricultural production [6].

Similarly, *Rhizoctonia solani* is a globally distributed soilborne pathogen that attacks seeds, roots, and stems across numerous crop species and is a major causal agent of damping-off and root rot; infections by *R. solani* can result in stand establishment failures and severe yield losses, including reductions in seed yield and plant vigor in affected crops.

In recent years, increasing attention has been directed toward the use of natural compounds as sustainable alternatives to synthetic fungicides. Among these, essential oils have attracted particular interest due to their well-documented antimicrobial and antifungal properties. Eugenol, a phenolic compound present in several essential oils, has demonstrated inhibitory activity against a wide range of phytopathogenic fungi, including *Penicillium expansum*, *P. glabrum*, *P. italicum*, *Aspergillus niger*, *Emericella nidulans*, *Aspergillus terreus*, and *Fusarium oxysporum* [7,8,9].

In this context, clove essential oil, which is rich in eugenol and other volatile constituents, has shown considerable potential as an antifungal agent [10,11,12]. Clove-derived products have also been successfully applied in the control of postharvest fungal diseases, further supporting their relevance in sustainable agricultural systems [13].

For instance, Muñoz Castellanos et al. reported that clove essential oil and its functional extracts exhibited significant in vitro antifungal activity against *Aspergillus niger* and *Fusarium oxysporum*, with effective concentrations ranging from 400 to 500 ppm after 10 days of incubation [12]. Moreover, in tomato-based applications, clove essential oil reduced the growth of *A. niger* by 50–70% and *F. oxysporum* by approximately 40% [12].

However, direct comparisons of the efficacy of pure eugenol versus clove oil against different plant pathogens remain limited. This study aims to evaluate and compare the antifungal activities of eugenol and clove oil against several phytopathogens, with the goal of identifying more effective and sustainable options for disease management in agricultural crop.

## 2. Results

### 2.1. Chemical Composition of Clove Essential Oil

A total of 67 compounds were identified by GC–MS analysis. Aromatic compounds, followed by sesquiterpene hydrocarbons (9.04%) and a smaller fraction of oxygenated sesquiterpenes (0.48%), dominated the composition based on relative area percentages obtained by GC/FID analysis. Within the aromatic category, the phenylpropanoid eugenol was the predominant component, accounting for 87.3% of the essential oil composition as determined by GC/FID. The remaining constituents were present only in trace amounts, highlighting their minimal contribution to the overall composition (Table 1).

### 2.2. Determination of the Antifungal Potential of Clove and Eugenol

**Figure 1:**
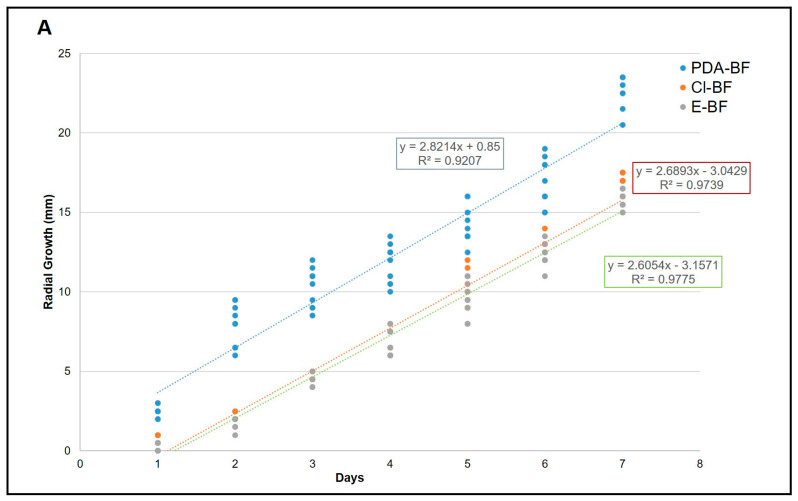

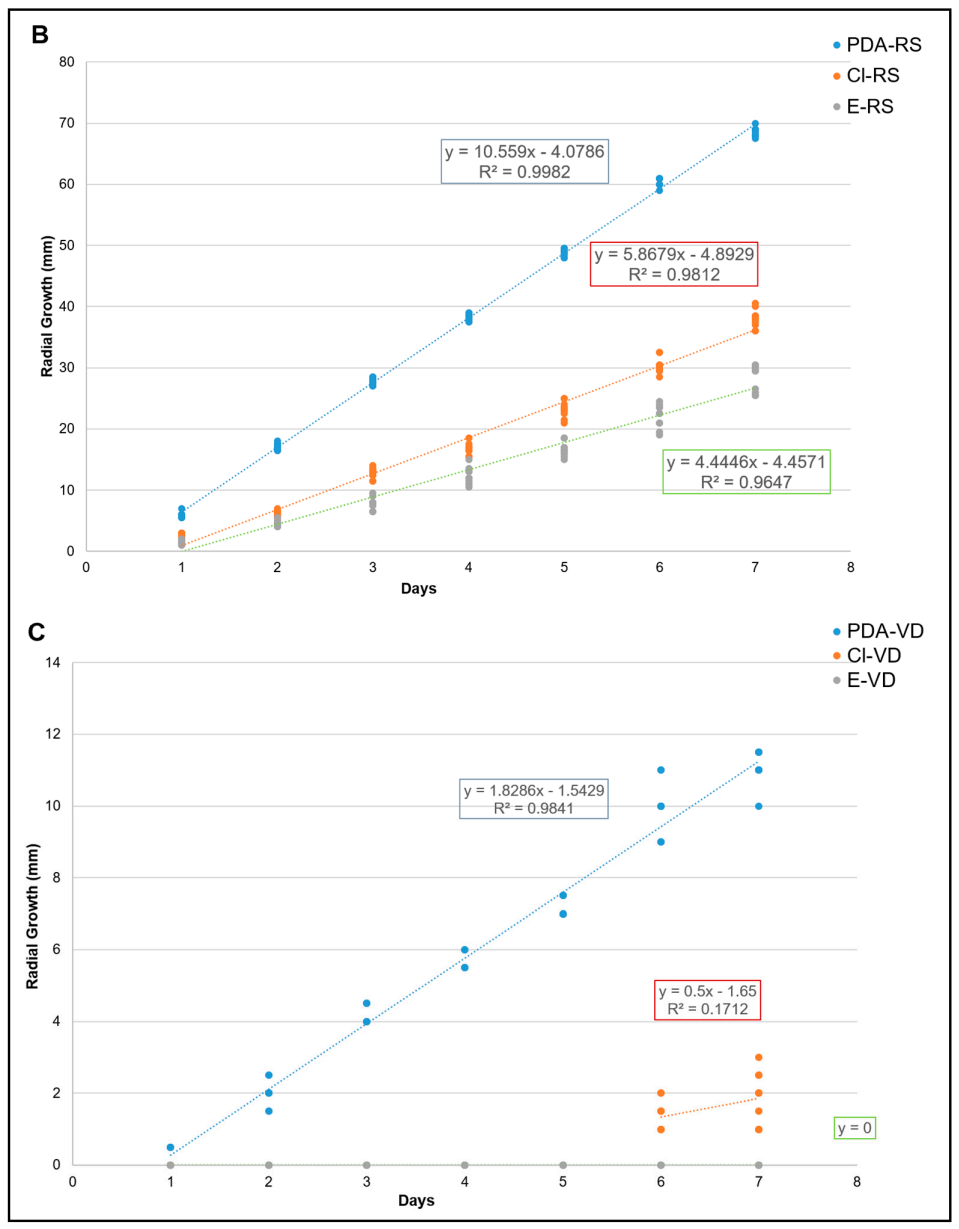
Growth rates (mm·day^−1^) of (**A**) *Botryotinia fuckeliana*, (**B**) *Rhizoctonia solani* and (**C**) *Verticillium dahliae* on clove essential oil (Cl) and pure eugenol (E) at 300 μg/mL, and potato dextrose agar (PDA).

The mycelial growth (expressed in mm) of *Botryotinia fuckeliana* (BF), *Rhizoctonia solani* (RS), and *Verticillium dahliae* (VD) was evaluated on PDA medium (control), PDA supplemented with *Syzygium aromaticum* essential oil (EO) at 300 μg/mL, and PDA with eugenol at 300 μg/mL. Growth ratios, mean growth rates, and standard deviations of the fungal species are summarized in Figure 1 and Table 2.

For *B. fuckeliana*, control growth was 43.00 ± 4.87 mm. The essential oil reduced this value to 35.50 ± 1.35 mm, while eugenol caused a more pronounced inhibition (31.50 ± 1.17 mm). *R. solani* exhibited very high control growth (137.20 ± 1.47 mm), which decreased to 76.00 ± 2.98 mm with the essential oil and to 56.70 ± 4.34 mm with eugenol. In *V. dahliae*, control growth was 22.20 ± 0.91 mm; the essential oil drastically reduced it to 3.70 ± 1.41 mm, and eugenol completely suppressed growth (0 mm).

From these data, the mycelial growth inhibition (MGI) percentage was calculated for each treatment, and the results are shown in Table 3. For *B. fuckeliana*, the essential oil achieved 44.61% inhibition, whereas eugenol reached 58.67%. For *R. solani*, the percentages were more moderate: 22.09% with the essential oil and 26.51% with eugenol. In contrast, *V. dahliae* showed high susceptibility, with 83.33% inhibition against the essential oil and complete inhibition (100%) in the presence of eugenol.

Overall, the results indicate that eugenol exhibits superior antifungal activity compared to *S. aromaticum* essential oil against the three species tested. The efficacy was especially notable in *V. dahliae*, whose growth was completely inhibited by eugenol. The low standard deviations observed in most treatments reflect good experimental reproducibility.

### 2.3. Tukey’s HSD Analysis of Fungal Growth Inhibition

A two-way ANOVA revealed significant effects of both fungal species and treatment on colony diameter. The fungal species factor showed a highly significant effect (SS = 102,747.0; df = 2; MS = 51,373.4; F = 7730.34; *p* < 0.0001), indicating substantial differences in growth among the three fungal species evaluated.

Likewise, treatment had a highly significant effect on fungal growth (SS = 23,995.0; df = 2; MS = 11,997.5; F = 1805.31; *p* < 0.0001), demonstrating that colony diameter differed significantly among the control, clove essential oil, and eugenol treatments.

Furthermore, a significant Species × Treatment interaction was detected (SS = 14,907.6; df = 4; MS = 3726.91; F = 560.80; *p* < 0.0001), indicating that the magnitude of the treatment effect depended on the fungal species tested. Therefore, the inhibitory activity of clove essential oil and eugenol was not consistent across all fungal species.

The residual error was low (MS = 6.65), indicating limited unexplained variability and good consistency among replicate measurements.

**Figure 2:**
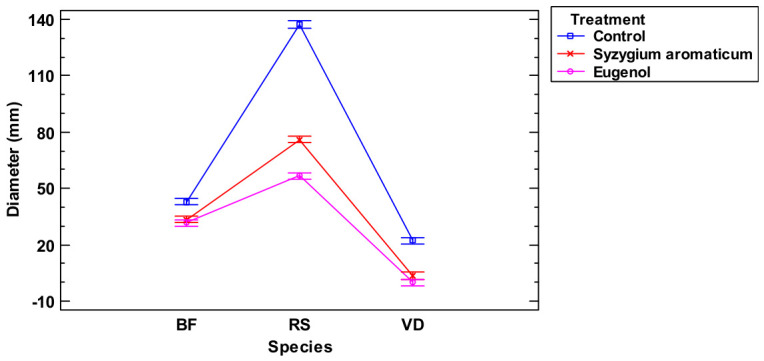
Interaction plot of mean fungal growth for control, clove essential oil, and eugenol (300 mg/L) against *Botryotinia fuckeliana* (BF), *Rhizoctonia solani* (RS), and *Verticillium dahliae* (VD). n = 30 observations per treatment.

Post hoc Tukey HSD tests revealed distinct response patterns among species. For *Botryotinia fuckeliana*, *Rhizoctonia solani*, and *Verticillium dahliae*, both clove essential oil and eugenol at 300 µg/mL significantly inhibited growth compared to the control, with statistically significant differences observed among all treatments. In contrast, although differences in growth were observed between clove essential oil and eugenol treatments, statistically significant differences were only detected for *Rhizoctonia solani* (Figure 2).

## 3. Discussion

The results of this study demonstrate the antifungal potential of clove essential oil and eugenol against three agriculturally relevant phytopathogens: *Botryotinia fuckeliana*, *Rhizoctonia solani*, and *Verticillium dahliae*.

Clove essential oil and its major constituent, eugenol, have been extensively investigated for their antifungal activity against a wide range of phytopathogenic fungi. Eugenol, which represents 87.3% of the oil in the present study, is a phenolic compound widely recognized for its ability to disrupt fungal cell membranes by increasing permeability and causing leakage of intracellular [7,14]. Moreover, eugenol interferes with ergosterol biosynthesis, a key component of fungal membranes, thereby compromising membrane integrity and function. It has also been reported to induce oxidative stress through the generation of reactive oxygen species (ROS), leading to damage to proteins, lipids, and nucleic acids [15,16].

However, despite the extensive literature on clove essential oil and eugenol individually, studies directly comparing the pure compound with the whole essential oil remain limited. Therefore, the present study provides novel insight by directly evaluating their relative efficacy, contributing to a better understanding of the relationship between chemical composition and antifungal activity.

The higher antifungal efficacy observed for pure eugenol compared to clove essential oil suggests that the biological effect of the oil cannot be attributed exclusively to the predominance of its major constituent. Although eugenol represents the main component of the essential oil, the lower activity observed for the complete mixture may indicate that minor constituents influence the overall antifungal activity through possible synergistic or antagonistic interactions. However, these interactions were not directly evaluated in the present study and therefore remain speculative.

In particular, sesquiterpenes such as β-caryophyllene and α-caryophyllene, as well as oxygenated derivatives such as caryophyllene oxide, may hypothetically exert secondary roles in modulating the physicochemical properties of the oil. Although these compounds have been reported to exhibit antimicrobial and, to a lesser extent, antifungal activity, their low abundance and lower chemical reactivity compared to phenolic compounds suggest a limited direct contribution to biological activity [17]. Instead, their presence may influence membrane partitioning processes and the distribution of more active constituents.

From a physicochemical perspective, these hydrophobic terpenes can integrate into lipid bilayers, altering membrane organization and potentially affecting the diffusion and availability of eugenol at the target site. Such interactions may modify compound–membrane affinity and overall bioactivity. This mechanistic interplay may therefore explain the reduced antifungal activity observed for the essential oil compared to pure eugenol, suggesting that its overall efficacy results from a balance between active and modulatory components rather than from the predominance of a single compound.

A limitation of the present study is that the comparative antifungal analysis was performed using a single concentration (300 μg/mL). Consequently, it remains unclear whether the relative differences observed between pure eugenol and clove essential oil would persist across a broader concentration range, including subinhibitory levels. This concentration was selected based on previous studies reporting relevant antifungal activity within this range [18].

In addition, the antifungal activity of a compound may vary considerably depending on the presence and interaction of other bioactive constituents. In this regard, previous studies evaluating compounds such as carvacrol, thymol, cinnamaldehyde, and eugenol against different fungal species have demonstrated marked concentration-dependent effects, including transitions between fungistatic and fungicidal activity depending on both dose and compound interactions. These findings highlight that antifungal activity and interactions among essential oil constituents are not constant and may vary according to concentration and chemical composition [19].

Further studies are needed to better elucidate the mechanisms underlying the interactions among the constituents of clove essential oil and their impact on antifungal activity. The results of the present study, showing higher efficacy of pure eugenol compared to the essential oil, suggest that such interactions may play a key role in modulating overall biological activity. In particular, experiments with isolated minor compounds such as β-caryophyllene and caryophyllene oxide, either alone or in combination with eugenol, would help determine whether their effects are synergistic, additive, or antagonistic.

Additionally, assessing their influence on fungal cell membrane integrity, ergosterol biosynthesis, and oxidative stress responses would provide deeper insight into their modes of action. Future research should also include a broader range of phytopathogenic species and explore different concentrations and formulations to better approximate agricultural conditions. Such studies would contribute to optimizing the use of clove-derived products as natural antifungal agents and to clarifying the relationship between essential oil composition and biological activity.

## 4. Materials and Methods

### 4.1. Essential Oil

The essential oil of *Syzygium aromaticum* was extracted by hydrodistillation, using 100 g of fresh flower buds in a Clevenger-type apparatus for a duration of 3 h. After extraction, the oil was collected and dried with anhydrous sodium sulfate (Fluka™, Buchs, Switzerland; purity ≥ 99.0%) to eliminate any remaining moisture. It was subsequently filtered using a 0.22 μm OlimPeak syringe filter (Teknokroma™, Barcelona, Spain) to remove particulate matter. The purified oil was then divided into aliquots, placed in amber glass vials, and stored at 4 °C to prevent photochemical degradation and oxidation prior to further analysis.

Sigma-Aldrich (St. Louis, MO, USA) supplied the pure eugenol.

### 4.2. Gas Chromatography (GC/FID)

The essential oil’s chemical composition was determined by gas chromatography with flame ionization detection (GC/FID) using a Perkin-Elmer Clarus 500GC instrument (PerkinElmer, Waltham, MA, USA). Compounds were separated on an HP-1 capillary column (30 m × 0.25 mm internal diameter, 0.25 µm film thickness). The oven temperature program started at 60 °C (held for 5 min), increased to 180 °C at 3 °C/min, and then rose to 280 °C at 20 °C/min, where it was maintained for 10 min. Helium served as the carrier gas with a constant flow rate of 1 mL/min. The injector and detector temperatures were set at 220 °C and 250 °C, respectively. Relative area percentages were obtained by area normalization (%).

### 4.3. Chromatography–Mass Spectrometry (GC–MS)

Component identification was carried out using gas chromatography–mass spectrometry (GC–MS) with a Varian Saturn 2000 system (Varian Inc., Palo Alto, CA, USA) fitted with a VA-5MS capillary column, matching the one used in the GC/FID analysis. The same operating conditions and oven temperature program were applied, with a split injection ratio of 1:25. The mass spectrometer functioned in electron impact mode at 70 eV, scanning a mass-to-charge (*m*/*z*) range of 28–400. Identification of the compounds was achieved by comparing their mass spectra and linear retention indices (LRIs), calculated using a homologous series of C8–C25 n-alkanes, with data from the NIST 2005 library, relevant literature [16], and authentic reference standards when available. Quantitative results (% relative composition) were obtained from the GC/FID analysis. Relative area percentages (%) were obtained from the GC/FID analysis.

### 4.4. Fungal Strains

Three phytopathogenic fungal strains—*Botryotinia fuckeliana* (BF, CECT 2100), *Rhizoctonia solani* (RS, CECT 2819), and *Verticillium dahliae* (VD, CECT 2694; risk group 1)—were supplied by the Spanish Type Culture Collection (CECT). Fungal cultures were maintained on potato dextrose agar (PDA) at 5 °C until use.

### 4.5. Antifungal Activity of Essential Oils

The antifungal activity was evaluated by incorporating the essential oil of clove or eugenol into the growth medium. Each compound was dissolved and homogenized in sterile potato dextrose agar (PDA) supplemented with 0.1% Tween 20 as an emulsifying agent, reaching a final concentration of 300 µg/mL. The amended media were then poured into sterile Petri dishes and allowed to solidify.

For inoculation, 8 mm diameter mycelial discs, obtained from the actively growing margins of 7-day-old colonies, were placed at the center of both treated and control plates. The plates were incubated at 25 °C for 7 days. Control plates consisted of PDA supplemented with 0.1% Tween 20 without the addition of antifungal compounds.

Fungal growth was assessed daily by measuring colony diameters in two perpendicular directions over a period of 7 days. In addition, growth rates (mm·day^−1^) were calculated by performing linear regression of colony radius (mm) as a function of time (days), allowing the estimation of radial growth dynamics for each treatment.

After incubation, fungal growth inhibition was determined by measuring final colony diameters, and the percentage of mycelial growth inhibition (MGI) was calculated. Each treatment was performed with thirty replicates.

The MGI (%) was calculated according to the method described by Albuquerque et al. [20].

MGI (%) = [(Dc − Do)/Dc] × 100

where:

Dc = average colony diameter in control plates (mm);

Do = average colony diameter in EO-treated plates (mm).

### 4.6. Statistical Analysis

The diameters of fungal colonies (n = 30 per group) measured under the different conditions—PDA (control), PDA supplemented with *Syzygium aromaticum* (300 μg/mL), and PDA supplemented with eugenol (300 μg/mL)—were tested for normality using the Shapiro–Wilk test. All datasets met the assumption of normality (*p* > 0.05) and homogeneity of variances (verified by Levene’s test, *p* > 0.05). The independence of observations was ensured through separate experimental units and randomized plate placement satisfying the prerequisites for parametric analysis

The antifungal activity data were analyzed using two-way analysis of variance (ANOVA) followed by Tukey’s Honestly Significant Difference (HSD) post hoc test for multiple comparisons between species and treatments. Statistical significance was established at *p* < 0.05. All analyses were performed using Statgraphics Centurion XIX (StatPoint Technologies, Inc., Warrenton, VA, USA).

## Figures and Tables

**Table 1:** Chemical composition (%) of the essential oil of *Syzygium aromaticum* (clove) determined by GC/FID.

RI ^a^	Compound ^b^	Clove % ^c^
794	propoanoic acid,2-methyl-,1-methylethyl ester ^b^	-
798	hexanal ^b^	-
847	butanoic acid,2-methyl-,ethyl ester ^b^	-
853	3-hexen-1-ol ^b^	-
888	isopropyl2-methylbutanoate ^b^	-
901	2-heptanol ^b^	-
918	butanoic acid,2-methylpropyl ester ^b^	-
932	α-thujene ^b^	-
939	α-pinene ^a,b^	-
950	2,4(10)-thujadien ^b^	-
954	camphene ^a,b^	-
979	sabinene ^a,b^	-
980	β-pinene ^a,b^	-
983	1-octen-3-ol ^b^	-
994	β-myrcene ^a,b^	-
995	3-octanone ^b^	-
1005	phellandrene ^b^	-
1013	δ-3-carene ^b^	-
1020	α-terpinene ^a,b^	-
1029	p-cymene ^a,b^	-
1033	limonene ^a,b^	-
1035	1,8-cineole (eucaliptol) ^a,b^	-
1063	γ-terpinene ^a,b^	-
1071	sabinene hydrate ^b^	-
1091	terpinolene ^b^	-
1095	2-nonanone ^b^	-
1001	linalool ^a,b^	-
1047	camphor ^a,b^	-
1165	pinocarvone ^a,b^	-
1166	benzene propanal ^b^	-
1168	acetic acid,phenylmethylester ^b^	-
1168	borneol ^a,b^	-
1180	terpinen-4-ol ^a,b^	-
1192	linalyl propionate ^b^	-
1193	α-terpineol ^a,b^	-
1199	estragole ^b^	-
1201	dihydrocarvone ^b^	-
1232	nerol ^b^	-
1259	chavicol ^b^	tr
1261	linalyl acetate ^b^	-
1273	2-propenal, 3-phenyl- ^b^	-
1288	bornyl acetate^b^	-
1289	safrole ^b^	-
1290	thymol ^a,b^	-
1296	2-undecanone ^b^	-
1298	carvacrol ^a,b^	-
1328	myrtenyl acetate ^b^	-
1357	α-terpinenyl acetate ^b^	-
1362	eugenol ^b^	87.3
1380	copaene ^b^	-
1409	methyl eugenol ^b^	-
1420	β-caryophyllene ^a,b^	6.90
1447	cinnamyl acetate ^b^	-
1453	α-caryophyllene ^a,b^	2.05
1475	β-cadinene ^b^	0.09
1478	γ-cadinene ^b^	tr
1486	β- selinene ^b^	tr
1494	α-selinene ^b^	tr
1499	α-muurolene ^b^	tr
1510	α-farnesene ^b^	tr
1519	δ-cadinene ^b^	-
1538	eugenyl acetate ^a,b^	-
1570	caryophyllenyl alcohol ^b^	tr
1582	caryophyllene oxide ^a,b^	0.40
1609	1,2-epoxide-humulene ^b^	0.08
1731	4-hydroxy-2-methoxycinnamaldehyde ^b^	0.08
1736	4-((1E)-3-hydroxy-1-propenyl)-2- methoxyphenol ^b^	tr
1936	phenol,3,5-diethyl- ^b^	-

^a^ Retention indices calculated against n-alkanes (C8-C30). ^a,b^, identification based on retention times of genuine compounds on the HP-5MS agilent column; ^b^ tentatively identified on the basis of computer matching of the mass spectra of peaks with the (Nist, Nist_msms, mainlib, replib, wiley7n) libraries. ^c^ Relative area percentages (%) were calculated from GC/FID data; tr, trace (<0.05%).

**Table 2:** Mean growth (mm) and standard deviation values calculation for each fungus species grown on PDA (control), PDA-*Syzygium aromaticum* EO and PDA-eugenol at 300 μg/mL.

Species	Control	*Syzygium aromaticum*	Eugenol
BF	43.00 ± 4.87	35.50 ± 1.35	31.50 ± 1.17
RS	137.20 ± 1.47	76.00 ± 2.98	56.70 ± 4.34
VD	22.20 ± 0.91	3.70 ± 1.41	0

BF: *Botryotinia fuckeliana*, RS: *Rhizoctonia solani*, VD: *Verticillium dahliae*.

**Table 3:** Mycelial Growth Inhibition (MGI) percentage for each fungus grown on PDA-*Syzygium aromaticum* EO, and PDA-eugenol at 300 μg/mL.

Species	*Syzygium aromaticum*	Eugenol
BF	44.61	58.67
RS	22.09	26.51
VD	83.33	100

BF: *Botryotinia fuckeliana*, RS: *Rhizoctonia solani*, VD: *Verticillium dahliae*.

## Data Availability

The original contributions presented in this study are included in the article. Further inquiries can be directed to the corresponding author.

## Abbreviations

ANOVA	Analysis of variance
BF	*Botryotinia fuckeliana*
CECT	Spanish Type Culture Collection
EO	Essential oil
GC–FID	Gas chromatography with flame ionization detection
GC–MS	Gas chromatography–mass spectrometry
HSD	Honestly significant difference
LRI	Linear retention index
LRIs	Linear retention indices
MGI	Mycelial growth inhibition
MS	Mean square
*m*/*z*	Mass-to-charge ratio
NIST	National Institute of Standards and Technology
PDA	Potato dextrose agar
ppm	Parts per million
ROS	Reactive oxygen species
RS	*Rhizoctonia solani*
SS	Sum of squares
VD	*Verticillium dahliae*
μg/mL	Micrograms per milliliter
°C	Degrees Celsius
α	Significance level
